# Incidence, Speciation, and Morpho-Genetic Diversity of *Penicillium* spp. Causing Blue Mold of Stored Pome Fruits in Serbia

**DOI:** 10.3390/jof7121019

**Published:** 2021-11-28

**Authors:** Aleksandra Žebeljan, Nataša Duduk, Nina Vučković, Wayne M. Jurick, Ivana Vico

**Affiliations:** 1 Faculty of Agriculture, University of Belgrade, 11000 Belgrade, Serbia; zheki24@gmail.com (A.Ž.); ninaradulovic@hotmail.com (N.V.); vico@agrif.bg.ac.rs (I.V.); 2 USDA-ARS, Food Quality Laboratory, Beltsville, MD 20705, USA; wayne.jurick@usda.gov

**Keywords:** postharvest decay, blue mold, *Penicillium expansum*, *Penicillium crustosum*, *Penicillium solitum*, pome fruit, fruit storage

## Abstract

Blue mold, caused by *Penicillium* spp., is one of the most economically important postharvest diseases of pome fruits, globally. Pome fruits, in particular apple, is the most widely grown pome fruit in Serbia, and the distribution of *Penicillium* spp. responsible for postharvest decay is unknown. A two-year survey was conducted in 2014 and 2015, where four pome fruits (apple, pear, quince, and medlar) with blue mold symptoms were collected from 20 storage locations throughout Serbia. Detailed morphological characterization, analysis of virulence in three apple cultivars, and multilocus phylogeny revealed three main *Penicillium* spp. in order of abundance: *P. expansum*, *P. crustosum*, and *P. solitum.* Interestingly, *P. expansum* split into two distinct clades with strong statistical support that coincided with several morphological observations. Findings from this study are significant and showed previously undocumented diversity in blue mold fungi responsible for postharvest decay including the first finding of *P. crustosum*, and *P. solitum* as postharvest pathogens of quince and *P. crustosum* of medlar fruit in the world, and *P. expansum* of quince in Serbia. Data from this study provide timely information regarding phenotypic, morphological and genotypic plasticity in *P. expansum* that will impact the design of species-specific detection tools and guide the development of blue mold management strategies.

## 1. Introduction

Pome fruits consist of apples (*Malus domestica* Borkh.), pears (*Pyrus communis* L.), quince (*Cydonia oblonga* Mill.), Asian pear (*Pyrus seratina* Rehd.), medlar (*Mespilus germanica* L.), and many other wild species of the *Rosaceae* family [1]. Most pome fruits are stored for extended periods of time (6 to 12 months) in a cold and controlled atmosphere. This allows fruit to be preserved and be of high quality so they can be available for year-round consumption and for trade to other countries. However, fruit rots reduce fresh fruit for consumption, negatively impact fruit quality, and contribute to mycotoxin contamination, specifically patulin, which is the case for *Penicillium* spp. [2].

Blue mold caused by *Penicillium* spp. is one of the most economically important postharvest diseases and a survey in Washington State revealed that it accounted for 28% of fruit decays in storage [3]. Blue mold is characterized by a soft, watery rot that is light brown in color accompanied by the appearance of blue-green conidia on the fruit surface that develops at advanced stages of decay. *P. expansum* Link., and other *Penicillium* spp. do not directly infect fruits, as they require wounds caused by stem punctures and bruises that occur before, during, and after harvest [2,4]. Conidia are the primary source of inoculum and are detectable in the packinghouse in flume water, on bin surfaces, fruit, and in the air [5,6,7,8]. Unfortunately, no resistance to blue mold is present in commercial apple cultivars as they are all susceptible [9].

*Penicillium expansum* is the most common and economically important causal agent of blue mold of stored apple and pear worldwide [9,10,11]. However, other species have been shown to be significant contributors of blue mold which include *P. aurantiogriseum* Dierckx, *P. brevicompactum* Dierckx, *P. carneum* Frisvad, *P. chrysogenum* Thom, *P. commune* Thom, *P. crustosum* Thom, *P. dendriticum* Pitt, *P. digitatum* (Pers.: Fr.) Sacc., *P. glabrum* (Wehmer) Westling, *P. griseofulvum* Dierckx, *P. ramulosum* Visagie & K. Jacobs, *P. rugulosum* Thom, *P. solitum* Westling and *P. verrucosum* Dierckx [5,6,12,13,14,15]. *P. expansum* has also been found to cause blue mold on quince [16]. However, it is well documented in the literature that *P. expansum* is the most common and aggressive species while *P. solitum* is a weak pathogen of apple and pears [6,17,18].

Apple is one of the most widely grown pomes in Serbia. In 2019, apples were cultivated on 26,089 ha, with a production of 499,578 tones, which comprises 3% of the total European apple production. Pear was produced on significantly less area, 4970 ha producing 54,859 t, representing 2.2% of the European pear market. Quince is a high value fruit in Serbia that is used to make jams, jellies, and brandy. Annual quince production is 11,074 t, from 1915 ha which provided 31% of total quince fruit grown in Europe. Serbia is the second largest quince producer in Europe and the ninth in the world [19]. *P. expansum* and *P. crustosum* have been described as causal agents of blue mold on apple [20,21,22,23] and pear fruit [24] in Serbia, and there are no data regarding incidence and distribution of blue mold fungal species on other pome fruits. Therefore, the aim of the current study was to identify the causal agents of blue mold on pome fruit in Serbia, gain insight into the diversity of *Penicillium* spp. via morphological and genetic characterization, and to evaluate the virulence of predominant species on different apple fruit cultivars.

## 2. Materials and Methods

### 2.1. Sample Collection and Fungal Isolation

**Figure 1:**
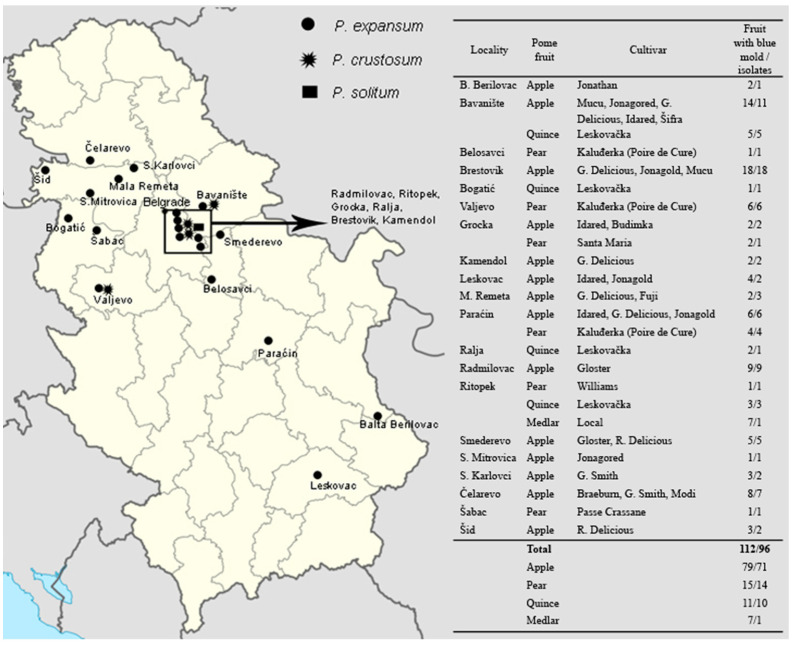
Map of Serbia showing different locations where *Penicillium* spp. isolates from pome fruits were obtained.

In a two-year survey, during 2014 and 2015, four different pome fruits (apple, pear, quince and medlar) with blue mold symptoms were collected from 20 storage locations (Figure 1). Fungal isolation was done from the internal portion of decayed tissue from surface disinfected fruit in 70% ethanol, after aseptic removal of the skin. Fragments from the margin of healthy and decayed tissue were placed on potato dextrose agar (PDA, EMD, Darmstadt, Germany, pH 5.6 ± 0.2) in Petri dishes (90 mm). After 5 days incubation at 24 ± 2 °C in the dark, colony fragments were transferred to PDA to obtain pure cultures, which were used to obtain monosporial isolates. For long term maintenance, isolates were kept as conidial suspensions in 30% glycerol 0.05% agar 0.05% Tween 20 (Sigma-Aldrich, Burlington, MA, USA) at −80 °C.

### 2.2. Pathogenicity Test

‘Idared’ apple, ‘Williams‘ pear, ‘Leskovačka‘ quince and medlar (local cultivar) fruit obtained from a local market (IPM orchard origin), were washed and surfaced sanitized with 70% ethanol, then wounded with the point of a finishing nail (14 mm × 4 mm) on two opposite sides of the fruit at the equator. Spore suspensions (~10^6^ spores/mL) were prepared by adding one fungal fragment (6 mm in diameter) from 7-day-old-cultures grown on PDA in 5 mL sterilized water with Tween 20 (0.05%) and adjusted using a haemocytometer [25,26]. Artificial inoculations were conducted by pipetting 40 µL of spore suspension into each wound and two fruit of every pome species were used per isolate. All isolates were inoculated onto apple fruit, whereas five isolates from pear were inoculated on pear, four isolates from quince onto quince and one from medlar onto medlar. Control fruit were wounded and inoculated with 40 µL of sterilized water containing Tween 20 (0.05%). Inoculated and control fruit were placed in plastic containers under high humidity and kept at 24 ± 2 °C for 7 days in natural light dark cycles. Re-isolation of the pathogen was performed as described above.

### 2.3. DNA Extraction, PCR Amplification, and Amplicon Sequencing

DNA extraction was performed by the CTAB protocol of Day and Shatock [27] from 7-day-old cultures of obtained 96 *Penicillium* spp. isolates grown on PDA (Supplementary Table S1). Initially, to detect presence of *P. expansum*, all isolates were tested using specific *P. expansum* primers PEF/PER, based on partial polygalacturonase gene (*Pepg*1), which amplifies a 404 bp fragment [28]. Further, for 26 selected isolates (including 18 detected *P. expansum* isolates selected based on pome fruit host, locality, cultivar, and difference in PDA colony reverse color, and 8 undetermined *Penicillium* isolates) nuclear ribosomal internal transcribed spacer region (ITS, 600 bp), portions of the β tubulin (*BenA*, 511 bp), calmodulin (*CaM*, 580 bp) and RNA polymerase II second largest subunit (*RPB2*, 1000 bp) were amplified using ITS1/ITS4, Bt2a/Bt2b, CMD5/CMD6 and 5F/7CR primers, respectively [29,30,31,32,33]. PCR reaction mix (25 µL) contained 1 µL of template DNA, 1xPCR Master Mix (Thermo Scientific, Vilnius, Lithuania) and 0.4 µM of each primer. Conditions for *Pepg*1 amplification were: initial denaturation at 92 °C for 5 min, followed by 30 cycles at 92 °C for 1 min, 55 °C for 45 s and 72 °C for 45 s, and final elongation at 72 °C for 10 min. Conditions for amplification of ITS region were: initial denaturation at 95 °C for 2 min, followed by 35 cycles at 95 °C for 30 s, 55 °C for 30 s and 72 °C for 60 s, and final elongation at 72 °C for 10 min. Conditions for *BenA* and *CaM* amplifications were: initial denaturation at 94 °C for 5 min, followed by 35 cycles at 94 °C for 45 s, 55 °C for 45 s and 72 °C for 60 s, and final elongation at 72 °C for 7 min. Conditions for *RPB2* amplification were: initial denaturation at 94 °C for 5 min, followed by 5 cycles at 94 °C for 45 s, 50 °C for 45 s and 72 °C for 60 s, then 5 cycles at 94 °C for 45 s, 52 °C for 45 s and 72 °C for 60 s and 30 cycles at 94 °C for 45 s, 55 °C for 45 s and 72 °C for 60 s. Final elongation occurred for 7 min at 72 °C [33]. PCR products (5 µL) were observed in 1.5% agarose gel, stained in ethidium bromide and visualized with UV transilluminator. Amplified products were purified and sequenced using forward and reverse primers to yield a 2X consensus amplicon sequence. Sequences were assembled using Pregap4 from the Staden programme package [34] and deposited in the NCBI GenBank.

### 2.4. Multilocus Sequence Analysis and Phylogeny

The obtained fungal DNA sequences were compared with those publicly available using MegaBlast (http://www.ncbi.nlm.nih.gov/, accessed on 1 June 2021). Related sequences and those of the closest species were retrieved from GenBank and aligned with those obtained in this study using ClustalX [35], under MEGA version X [36]. Evolutionary history was inferred based on individual and combined analyses of four loci (ITS, *BenA*, *CaM*, and *RPB2*) of 26 isolates obtained in this study, reference isolates and *Penicillium lanosocoeruleum* CBS 215.30 as an outgroup (Table 1), using the Maximum Likelihood (ML) and Maximum Parsimony (MP) methods (MEGA X). For ML, the best nucleotide substitution model was determined using the “find best model” option in MEGA X. Initial tree(s) for the heuristic search were obtained automatically by applying Neighbour-Join and BioNJ algorithms to a matrix of pairwise distances estimated using the Maximum Composite Likelihood approach, and then selecting the topology with superior log likelihood value. The MP trees were obtained using the Subtree-Pruning-Regrafting (SPR) algorithm with search level 3, in which the initial trees were obtained by the random addition of sequences (ten replicates). To estimate the statistical significance of the inferred clades, 1000 bootstraps were performed.

### 2.5. Cultural Morphology

For 26 selected isolates, colony morphology (appearance, presence of exudate, reverse color) and growth were analysed on Malt Extract Agar (MEA), Czapek Yeast Extract Agar (CYA), and Yeast Extract Sucrose Agar (YES) as described by Visagie et al. [33], and additionally on Potato Dextrose Agar (PDA). Plates were inoculated at three points with 1 µL of spore suspension (10^6^ conidia/mL, as stated for pathogenicity test) of each isolate. Three plates were used per media. All inoculated plates were kept in the dark at 24 ± 2 °C. Morphology of conidiophores and conidia, type of conidiophores and their ornamentation were evaluated from 7–10 day-old-cultures grown on MEA at 24 ± 2 °C [18,33] using compound microscope Zeiss Axio Lab, Jena, Germany. Photographs of conidiophores and conidia and their sizes were obtained using camera: Axiocam ERc 5s, Zeiss and software ZEN 2 (blue edition), Jena, Germany.

### 2.6. Ehrlich Test

The production of cyclopiazonic acid and other alkaloids was examined according to Lund [37]. Filter paper (20 mm × 20 mm) was immersed in Ehrlich reagent (2 g 4-dimethylamino-benzaldehide in 85 mL 96% ethanol, with 15 mL 10 N HCl) and was placed on top of the mycelial side of agar plugs (three plugs per isolate, diameter 8 mm) from 7 day-old-cultures grown on CYA at 24 ± 2 °C. After 2–10 min incubation, color or color change were recorded.

### 2.7. Virulence Assessment in Apple Fruit

Fungal virulence was assessed using 15 selected isolates of *Penicillium* spp. (seven *P. expansum*, seven *P. crustosum* and one *P. solitum* isolate) (Table 1) on three apple cultivars: ’Golden Delicious’, ’Red Delicious’, and ’Granny Smith’. Mature apple fruit were wound-inoculated as described in the pathogenicity test. Each apple fruit was inoculated with 40 µL of spore suspension on two opposite sides of the fruit. Spore suspensions were adjusted to concentration 10^5^ conidia/mL using a haemocytometer [25,26]. Two apple fruit were used per cultivar (six per isolate). Control fruit were wounded and inoculated containing 40 µL of sterilized water with Tween 20 (0.05%). All fruit were kept under moist conditions in sterile plastic boxes at 24 ± 2 °C temperature under natural light dark cycles. Lesion size was measured at the equatorial and longitudinal axes at 7- and 9-days post inoculation (dpi).

### 2.8. Statistical Analysis

Kolmogorov–Smirnov and Shapiro–Wilk tests were used to check for normal distribution of the data and Levene test assessed homogeneity of variances. Some of the data were not normally distributed, and homogeneity of variances was violated. Due to large sample sizes parametric tests were used [38,39]. To avoid type I error, we chose more stringent conditions by using *p* = 0.01 as a significance level. Two-way ANOVA was used to determine which species and culture media affected colony size, and how species and cultivar impacted lesion diameter. One-way ANOVA was used to determine if there was a difference in colony size between isolates, species or between culture media; difference in sizes of conidiophore parts (ramus, metulae, phialide and conidia) between isolates or species and to determine a difference in lesion size between isolates, species or cultivars. Post hoc Tukey HSD test was used to evaluate differences that occurred in testing multiple groups (between isolates of same species or between three species). Evaluating effect size of two factors on the dependent variable was measured using partial eta squared (η^2^). η^2^ < 0.06 is considered as weak; η^2^ > 0.06 moderate and η^2^ > 0.14 as strong effect [40]. The statistical analyses were conducted with IBM SPSS 22 software (SPSS Inc., Chicago, IL, USA).

### 2.9. Cluster Analysis

To interpret the similarities and differences amongst *P. expansum* or *P. crustosum* isolates, a multivariate statistical analysis with hierarchical cluster analysis based on Ward’s method and Euclidian distance interval was used. To prevent dominance by large values at the expense of small ones, data were *z*-standardized using the formula: *z* = (*x* − *μ*)*σ* − 1. IBM SPSS Statistics 22 (SPSS Inc., Chicago, IL, USA), was used for statistical analysis.

### 2.10. Principle Component Analysis

To enable a comprehensive assessment of data obtained in this study, a Principal Component Analysis (PCA) with PAST 3.17 software [41] was used. To prevent dominance in the PCA by large values at the expense of small ones, data were *z*-standardized using the formula: *z* = (*x* − *μ*)*σ* − 1. Morphological characteristics (colony diameter on all tested media, conidia, metulae, and rami width, and metulae length, presence of yellow pigment in PDA and MEA reverse, and CYA reverse color) were analysed for 26 isolates, while both morphology and virulence were assessed for 14 select isolates.

**Table 1:** Isolates used in this study.

Isolate	Geographic Origin/Host cv.	Date	GenBank Accession Number				References
			ITS	*BenA*	*CaM*	*RPB2*	
*Penicillium expansum*							
JRad4	Serbia, Radmilovac/Apple ‘Gloster’	December 2014	MZ364021	MZ364047	MZ364097	MZ364075	This study
3JC6	Serbia, Čelarevo/Apple ‘Braeburn’	January 2015	MZ364023	MZ364056	MZ364098	MZ364076	This study
3JC11	Serbia, Čelarevo/Apple ’Granny Smith’	April 2015	OK432548	MZ364048	MZ364099	MZ364077	This study
3JC23	Serbia, Čelarevo/Apple ‘Modi’	April 2015	MZ364024	MZ364049	MZ364100	MZ364078	This study
3JB13	Serbia, Brestovik/Apple ’Jonagold’	January 2015.	MZ364025	MZ364050	MZ364101	MZ364079	This study
3JB22	Serbia, Brestovik/ Apple ‘G. Delicious’	January 2015	MZ364026	MZ364061	MZ364102	MZ364080	This study
3SD3	Serbia, Smederevo/Apple ‘R. Delicious’	January 2015	MZ364027	MZ364051	MZ364103	MZ364081	This study
3SD5	Serbia, Smederevo/Apple ‘R. Delicious’	January 2015	MZ364028	MZ364052	MZ364110	MZ364082	This study
3S1	Serbia, Šid/Apple ‘R. Delicious’	January 2015	MZ364029	MZ364057	MZ364104	MZ364083	This study
3MR1	Serbia, Mala Remeta/Apple ‘G. Delicious’	January 2015	MZ364030	MZ364053	MZ364105	MZ364084	This study
KSA5	Serbia, Šabac/Pear ‘Passe Crassane‘	March 2015	MZ364031	MZ364045	MZ364111	MZ364071	This study
JMR2o *	Serbia, Mala Remeta/Apple ‘Fuji’	November 2015	MZ364033	MZ364058	MZ364108	MZ364085	This study
JMR2z *	Serbia, Mala Remeta/Apple ‘Fuji’	November 2015	MZ364034	MZ364059	MZ364109	MZ364086	This study
JBA8b *	Serbia, Bavanište/Apple ‘Jonagored‘	December 2015	MZ364035	MZ364054	MZ364106	MZ364087	This study
JPN2 *	Serbia, Paraćin/Apple ‘Idared’	December 2015	MZ364036	MZ364055	MZ364107	MZ364088	This study
KPN4 *	Serbia, Paraćin/Pear ‘Poire de Cure‘	December 2015	MZ364032	MZ364046	MZ364112	MZ364072	This study
DBA5 *	Serbia, Bavanište/Quince ‘Leskovačka’	December 2015	MZ364022	MZ364062	MZ364114	MZ364073	This study
DRI4a *	Serbia, Ritopek/Quince ‘Leskovačka’	December 2015	MZ364020	MZ364060	MZ364113	MZ364074	This study
*Penicillium crustosum*							
JBA8a *	Serbia, Bavanište/Apple ‘Jonagored’	December 2015	MZ364037	MZ364067	MZ389067	MZ364089	This study
JBA11 *	Serbia, Bavanište/Apple ‘Šifra’	December 2015	MZ364038	MZ364068	MZ389061	MZ364090	This study
KGR2 *	Serbia, Grocka/Pear ‘Santa Maria’	October 2015	MZ364039	MZ364063	MZ389062	MZ364091	This study
KRI1P *	Serbia, Ritopek/Pear ‘Williams’	December 2015	MZ364040	MZ364064	MZ389063	MZ364092	This study
KVA8 *	Serbia, Valjevo/Pear ‘Poire de Cure‘	December 2015	MZ364041	MZ364065	MZ389064	MZ364093	This study
DRI4b *	Serbia, Ritopek/Quince ‘Leskovačka’	December 2015	MZ364042	MZ364066	MZ389065	MZ364094	This study
MRI4 *	Serbia, Ritopek/Medlar local cv.	December 2015	MZ364043	MZ364069	MZ389066	MZ364095	This study
*Penicillium solitum*							
DRI3 *	Serbia, Ritopek/Quince ‘Leskovačka’	December 2015	MZ364044	MZ364070	MZ364115	MZ364096	This study
**NCBI isolate**							
*Penicillium expansum*							
CBS 325.48 **	USA/Apple fruit		AY373912^1^	AY674400^2^	DQ911134^2^	JF417427^2^	^1^[42]; ^2^[43]
F758	USA, Idaho/ Sugar beet	2014	MG714838	MG714864	MG714821	MG714845	[44]
PCAS	Italy/Chestnut	2015	MG821365	MF100860	MF100880	/	[45]
P34	Italy /Withered grape		/	KU554673	/	/	[46]
4	Greece/Kiwi fruit	2017	/	MH040784	/	/	[47]
CV 2860	South Africa/Fynbos biome		/	JX091539	JX141580	/	[48]
CV 2861	South Africa/Fynbos biome		/	JX091540	JX141581	/	[48]
LUB	Serbia, Ub/Onion	2015	/	KY770971	/	/	[49]
*Penicillium marinum*							
CBS 109550 **	Japan/Sandy soil		KJ834512	AY674392	KU896842	KU904357	[42]
CBS 109547	Tunisia/Sandy soil		/	AY674390	/	/	[50]
*Penicillium crustosum*							
CBS 115503 **	Scotland, Aberdeen/Lemon		MH862985^1^	AY674353^2^	DQ911132^3^	MN969114^3^	^1^[51]; ^2^[50]; ^3^[42]
CV 0241	South Africa/Fynbos biome		JX091403	JX091536	JX141576	MN149972	[52]
5A	Italy/Chestnut	2015	MG821363	MF100874	MF100894	/	[45]
N2AS	Serbia/Nectarine	2021	/	MT799805	/	/	[53]
*Penicillium echinulatum*							
CBS 317.48 **	Canada, Culture contaminant		AF033473	AY674341	DQ911133	KU904352	[42]
DTO228I4	Unknown/Unknown		/	MN149925	MN149944	MN149964	[52]
*Penicillium solitum*							
CBS 424.89 **	Germany/Unknown		AY373932^1^	AY674354^2^	KU896851^1^	KU904363^1^	^1^[42]; ^2^[50]
XF	Italy/Chestnut	2015	MG821373	MF100861	MF100881	/	[45]
*Penicillium discolor*							
CBS 474.84 **	Israel/*Raphanus sativus*		AJ004816	AY674348	KU896834	KU904351	[42]
DTO047A2	Unknown/Unknown		/	MN149922	MN149941	MN149961	[52]
*Penicillium lanosocoeruleum*							
CBS 215.30	USA/Culture contaminant		NR_163541^1^	KU896817^2^	JX996967^2^	JX996712^3^	^1^[51]; ^2^[42]; ^3^[54]

* Isolates used in virulence study; ** Ex-type/referent isolates.

## 3. Results

### 3.1. Blue Mold Symptoms and Koch’s Postulates

**Figure 2:**
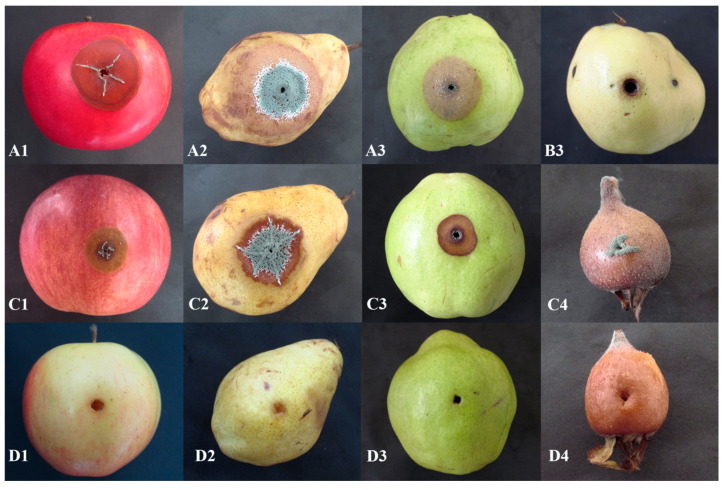
Blue mold on pome fruits: apple (1), pear (2), quince (3), and medlar (4) inoculated with representative isolates of *P. expansum* (**A**), *P. solitum* (**B**), *P. crustosum* (**C**), and uninoculated control (**D**).

Pome fruits, sampled from 20 different locations in Serbia, exhibited typical blue mold symptoms. The decayed area was light brown, soft and watery, and easily separated from the healthy tissue, and ranged from small lesions to completely decayed fruit. In most cases decayed fruit were covered with blue-green colored spores, and had an earthy, musty odour. In total, 96 *Penicillium* spp. isolates were obtained, 71 originating from apples, 14 from pears, 10 from quince, and one from medlar. All isolates were pathogenic and caused decay on inoculated healthy ‘Idared’ apple fruit, whereas five isolates from pear were pathogenic to pear, four isolates from quince to quince and one from medlar to medlar. *P. crustosum* induced slightly darker colored decay on inoculated pome fruits than *P. expansum*, which was the most evident on fruits with yellow skin. Control fruits remained symptomless. On inoculated fruits, blue-green sporulation was mainly present at the inoculation site. Reisolated fungi exhibited identical morphological characteristics that mirrored the original isolate, thus completing Koch’s postulates. Blue mold on representative inoculated pome fruits is presented in Figure 2.

### 3.2. Molecular Identification and Single Nucleotide Polymorphism

*P. expansum* specific primers (PEF/PER) generated amplicons of 404 bp in 88 out of 96 tested isolates, while no amplification was observed in 8 isolates and the negative controls. For ITS, *BenA*, *CaM*, and *RPB2* amplicons of expected size (600, 511, 580, 1000 bp, respectively) were obtained in 26 selected isolates (18 isolates which were positive with *P. expansum* specific primers and 8 isolates other than *P. expansum*). Sequencing of the obtained amplicons yielded nucleotide sequences 559–561 nt long for ITS, 428–432 nt for *BenA*, 501–504 nt for *CaM*, and 971 nt for *RPB2*, excluding primers, which were deposited in the NCBI GenBank under accession numbers given in Table 1.

The identity of isolates was confirmed based on single and multilocus phylogeny. Therefore, 18 isolates were identified as *P. expansum* and confirmed the results of specific primer identification, while seven isolates were identified as *P. crustosum*, and one as *P. solitum*. Molecular identification (total of 96 *Penicillium* isolates) revealed that 91.64% of blue mold on pome fruit collected in this study was caused by *P. expansum*, 7.29% *P. crustosum* and 1.04% *P. solitum*.

Multiple sequence alignment of the *P. expansum* isolates revealed that: (i) no sequence variation was identified in ITS amplicons, (ii) two sequence variants were found in *BenA* sequences (2 nt differences) and *CaM* (3 nt differences), and (iii) three sequence variants were identified in *RPB2* (1, 14 and 15 nt differences) (Table 2). *Penicillium expansum* ITS sequences were identical to those from *P. expansum* deposited in NCBI GenBank (i.e., AY373912, MG714838, and MG821365). One *BenA* variant (11 isolates) had sequences that matched several *P. expansum* sequences (i.e., JX091539, JX091540) and the other *BenA* variant (7 isolates) had sequences in line with *P. expansum* (i.e., MH040784) deposited in NCBI. One *CaM* variant (11 isolates) was identical with *P. expansum* sequences deposited in NCBI GenBank (e.g., DQ911134, MG714821). The other *CaM* variant (7 isolates) differed in 3 nt. One *RPB2* variant (12 isolates) was identical with *P. expansum* sequences JF417427 and MG714845, while the second *RPB2* variant (four isolates) showed 14 nt differences, and the third (two isolates) had 1 nt difference. Between second and third *RPB2* variants, the difference was in 15 nt (Table 2).

Multiple sequence alignment of the *P. crustosum* isolates revealed that: (i) no sequence variation was identified in sequences of ITS and *BenA*, (ii) two sequence variants occurred in *CaM* genomic fragments (1 nt differences), and iii) four variants were identified in *RPB2* (having 1, 1, 1, 2 nt differences) (Table 2). ITS and *BenA* from *P. crustosum* sequences (seven isolates) were identical with those from *P. crustosum* isolate CV 0241, i.e., JX091403 and JX091536, respectively. Based on the *CaM* locus, six isolates were identical, and matched sequences of the *P. crustosum* isolate CV 0241 (JX141576) and CBS 115503 (DQ911132), while the sequence of one isolate, JBA8a differed from the others in 1 nt. One *RPB2* variant (two isolates) was identical with the sequence from *P. crustosum* isolate CBS 115503 (MN969114), while the second *RPB2* variant (two isolates) matched CV 0241 (MN149972), and the variants differed in 2 nt. The third *RPB2* variant (two isolates) differed in 2 nt from both the first and the second variant. The fourth variant (one isolate) differed in 3, 3, and 1 nt from the first, second, and third variant, respectively.

*P. solitum* obtained ITS and *CaM* sequences were identical with those of *P. solitum* CBS 424.89 (AY373932 and KU896851, respectively) and *BenA* had the highest similarity with CBS 424.89 (AY674354), while *RPB2* aligned with *P. solitum* CBS 424.84 (KU904363).

### 3.3. Macromorphology

**Figure 3:**
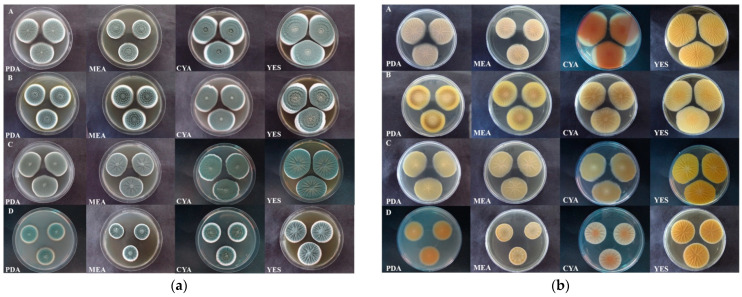
Colony characteristics (**a**) and colony reverse (**b**) of representative *Penicillium expansum* (A—group I, B—group II), *P. crustosum* (C), and *P. solitum* (D) isolates on different media (PDA, MEA, CYA, and YES) 7 dpi at 24 ± 2 °C.

*P. expansum* isolates formed white mycelia (visible as white margins) with blue green conidia on all media. On PDA, colonies were mostly fasciculate with or without concentric zones and radially sulcate. Colony margins were entire. Exudate was absent. Colony reverse in most isolates was cream to pale yellow except for isolates 3JC6, 3S1 (intense yellow with brown ring around centre), KSA5 (intense yellow with cream centre), and JMR2z (dark yellow). On MEA, *P. expansum* formed fasciculate colonies mostly with concentric zones and were radially sulcate. Scarce clear exudate droplets were present on the colony edge. Colony reverse was in most isolates cream coloured, while in some yellow (3JC6, KSA5, and JMR2z—intense yellow with cream centre; 3S1—yellow with light brown ring). On CYA, colonies were concentrically fasciculate and weakly radially sulcate, rarely velutinous. Colonies were dense and differed in exudate production from no exudate, scarce, to abundant exudate droplets. Colony reverse varied from cream, yellow, salmon pink to red. Most isolates formed salmon pink to red reverse of different intensities, some formed yellow reverse (3JC6, 3S1, and KSA5) and some cream reverse (3JB22, JMR2o, and DBA5). On YES, colonies were mostly fasciculate with or without concentric zones, radially sulcate, sometimes velutinous (3JC23, KPN4, and DRI4a). Conidia color was blue green to grey, and colonies were sometimes cream around the centre, and exudate was absent. Colony reverse was pale yellow to intense yellow, with or without orange centre (Figure 3a,b).

*P. crustosum* isolates formed white mycelia (visible as white margins) with blue dull green conidia on all media. Colonies on PDA and MEA were similar: velutinous, sometimes radially sulcate, which become crustose after 10 days. Colony margins were white and exudate was absent. Colony reverse was cream to intense yellow. On CYA, *P. crustosum* formed velutinous, sometimes radially sulcate colonies mainly with irregular margins. Colonies were with or without exudate. Colony reverse was cream to yellow. On YES, colonies were velutinous, radially sulcate and dense. Conidia color was blue green and sometimes grey. Margins were entire and exudate was absent. Colony reverse was yellow to intense yellow (Figure 3a,b). Among *P. crustosum* one isolate (DRI4b) differed by forming concentrically floccose to fasciculate colonies on CYA and YES, and orange reverse on PDA, MEA, and CYA.

*P. solitum* isolate formed white mycelia with blue green conidia on all media. On PDA, colony was velutinous to floccose with concentric zones, without exudate. Colony reverse was orange with a cream margin. On MEA, the colony was floccose and radially sulcate, without exudate. Colony reverse was cream to light brown at the margin. On CYA, colony was floccose and radially sulcate, and abundant clear exudate droplets were present. Colony reverse was cream with a bright orange centre. On YES, colony was floccose and radially sulcate with bright blue grey conidia. Exudate was absent. Colony reverse was intense yellow (Figure 3a,b).

### 3.4. Colony Growth

**Figure 4:**
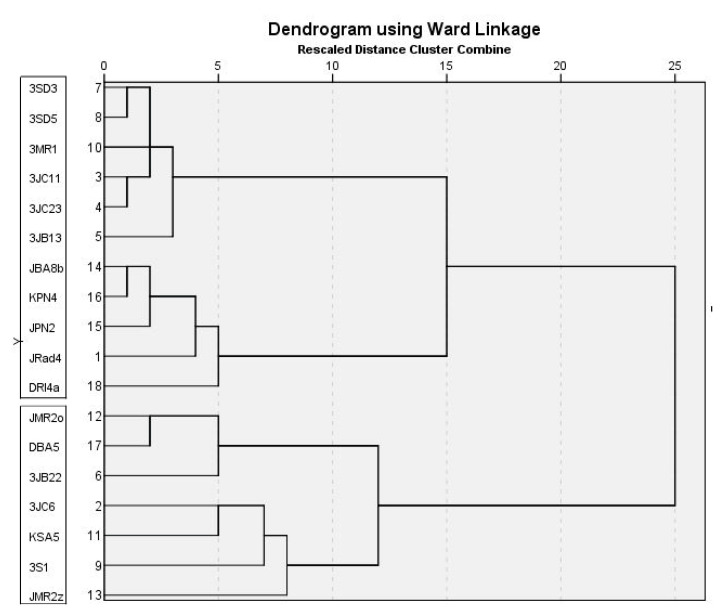
Dendogram showing the grouping of *Penicillium expansum* isolates based on colony diameter on different media (PDA, MEA, CYA, and YES), reverse on PDA and MEA (cream or yellow), and CYA reverse characteristics, using Ward’s model with Euclidian Distance interval. *P. expansum* isolates within a clade are indicated in black boxes.

**Figure 5:**
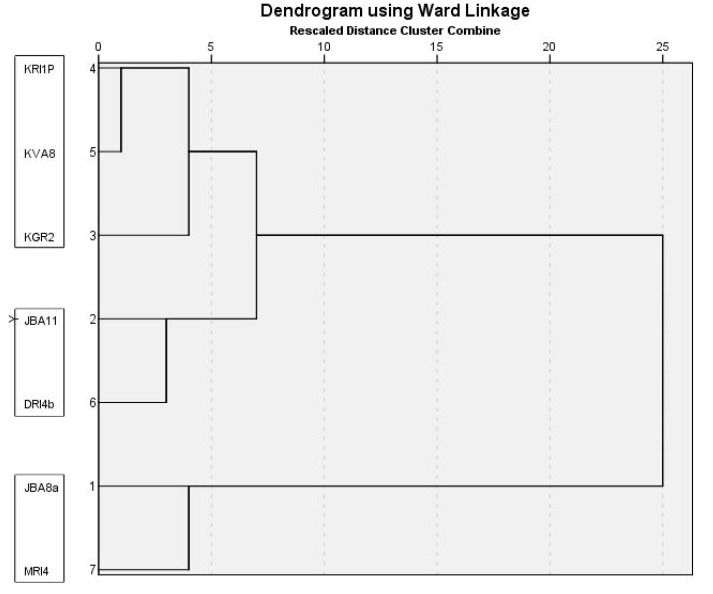
Dendogram showing grouping of *Penicillium crustosum* isolates based on colony diameter on different media (PDA, MEA, CYA, and YES), using Ward’s model with Euclidian Distance interval. *P. crustosum* isolates within a clade are indicated in black boxes.

Colony growth of *P. expansum*, *P. crustosum* and *P. solitum* on four media is presented in Table 3. The most favourable growth media for all three species was YES, followed by CYA and PDA, while colony diameter was smaller on MEA (*p* < 0.01). *P. expansum* growth was faster on CYA and YES compared to *P. crustosum* (*p* < 0.0001) (and *P. solitum* (*p* < 0.0001)), while on PDA and MEA was the same as *P. crustosum* (*p* = 0.901). *P. solitum* had the slowest colony growth on all media (*p* < 0.01). Variability in colony growth was observed within isolates of *P. expansum* (on all media) (*p* < 0.01) and *P. crustosum* (on MEA, CYA, and YES) (*p* < 0.01). Among *P. expansum* two groups of isolates were observed, fast growing (11 out of 18) and slower growing (7 out of 18) (Table 3, Figure 4). Two-way ANOVA revealed that both species and culture media had a large influence on colony growth. Variation in colony growth of 20.9% (η^2^ = 0.209, *p* < 0.0001) is due to differences between species, while 17.3% (η^2^ = 0.173, *p* < 0.0001) is caused by differences in culture media. Combined effect of species characteristics and culture media on its growth was negligible 2.6% (η^2^ = 0.026, *p* < 0.0001). We did not observe that isolate origin had an influence on colony growth (data not shown). Cluster analysis based on colony diameter on three different media (PDA, CYA, and YES), presence/absence of yellow colony reverse on PDA and MEA, and colony reverse on CYA separated *P. expansum* isolates into two groups and corresponds with two sub clusters obtained via phylogenetic analysis (*BenA*, *CaM*) (Figure 4. The first group of isolates (11 out of 18) had faster growing colonies, cream reverse (absence of yellow reverse) on PDA and MEA, and salmon pink to red CYA reverse. A second group of isolates (7 out of 18) had slower colony growth, yellow reverse on PDA and MEA, and variable color of CYA colony reverse (yellow, cream or red). Cluster analysis based on colony growth on three different media (MEA, CYA, and YES) separated *P. crustosum* isolates into three groups (Figure 5), and it corresponds with three sub clusters obtained via phylogenetic analysis (*RPB2* and multilocus).

### 3.5. Micromorphology

*P. expansum* formed mostly terverticillate and sometimes biverticillate conidiophores with smooth walls. Conidia were grey green to blue green in color, smooth, globose, subglobose, and elliptical, with average conidia size 3.24 ± 0.30 × 2.73 ± 0.24 µm. Phialides were smooth, mostly cylindrical and sometimes ampuliform, with average size 10.14 ± 1.32 × 2.94 ± 0.47 µm. Metulae (12.94 ± 2.30 × 3.61 ± 0.65 µm) and rami (19.66 ± 4.47 × 3.96 ± 0.97 µm) were smooth and cylindrical. *P. crustosum* formed mostly terverticillate and sometimes biverticillate conidiophores with evidently roughened walls. Conidia were dull green to blue green, smooth, globose to subglobose, with average conidia size 3.33 ± 0.27 × 3.06 ± 0.23 µm. Phialides were smooth, rarely roughened, ampuliform to cylindrical, and its average size was 10.05 ± 1.35 × 3.10 ± 0.41 µm. Metulae were roughened to smooth and cylindrical (14.02 ± 2.43 × 3.86 ± 0.54 µm), while rami were roughened and cylindrical (21.23 ± 4.45 × 4.11 ± 0.60 µm). Stipes were also roughened. *P. solitum* formed mostly terverticillate and sometimes biverticillate conidiophores with smooth or finely roughened walls. Conidia were smooth, mostly globose sometimes subglobose, with average size 3.67 ± 0.17 × 3.42 ± 0.18 µm. Color of conidia was grey green to blue green, the same as *P. expansum* conidia. Phialides were smooth, ampuliform to cylindrical, with average size 10.13 ± 1.92 × 3.11 ± 0.41 µm. Metulae were smooth and cylindrical (12.20 ± 2.03 × 4.21 ± 0.56 µm), while rami were smooth to finely roughened and cylindrical (18.17 ± 4.09 × 4.41 ± 0.69 µm). Stipes were roughened. Comparative micromorphology (Table 4) between *P. expansum* and *P. crustosum* revealed: (i) there was no difference in conidia length (*p* = 0.09), while conidia of *P. expansum* were smaller in width than *P. crustosum* (*p* < 0.0001) (*P. solitum* isolate had significantly larger conidia than *P. expansum* and *P. crustosum* (*p* < 0.0001); (ii) there was no difference in phialides length (*p* = 0.75), and width (*p* = 0.011); (iii) metulae length and width were larger in *P. crustosum* compared to *P. expansum* (*p* < 0.0001) (*P. solitum* formed the widest metulae); (iv) rami were of similar size (*p* = 0.368 for length, *p* = 0.177 for width) (*P. solitum* formed the shortest and widest rami); (v) difference between investigated species was large in conidia width (η^2^ = 0.175), moderate in conidia length (η^2^ = 0.061), small in metulae and rami length and width (η^2^ = 0.047; η^2^ = 0.055 for metulae and η^2^ = 0.03; η^2^ = 0.023 for rami), (vi) variability within *P. expansum* isolates was observed in conidia, phialide, metulae, and rami size, while within *P. crustosum* isolates in conidia size and phialide length (*p* < 0.01). Isolate origin did not impact conidia or conidiophore size (statistical data not shown).

### 3.6. Ehrlich Test

Ehrlich reagent reacted with 12 out of 18 *P. expansum* isolates as yellow rings were detected and six isolates formed faint violet rings. All *P. crustosum* isolates formed faint yellow to yellow rings and *P. solitum* isolate also formed a yellow ring. Observed reactions indicated that six *P. expansum* isolates produced cyclopiazonic acid, while the other 12 *P. expansum* isolates, and all *P. crustosum* isolates and *P. solitum* isolate produced other alkaloids.

### 3.7. Penicillium Multilocus Phylogeny

**Figure 6:**
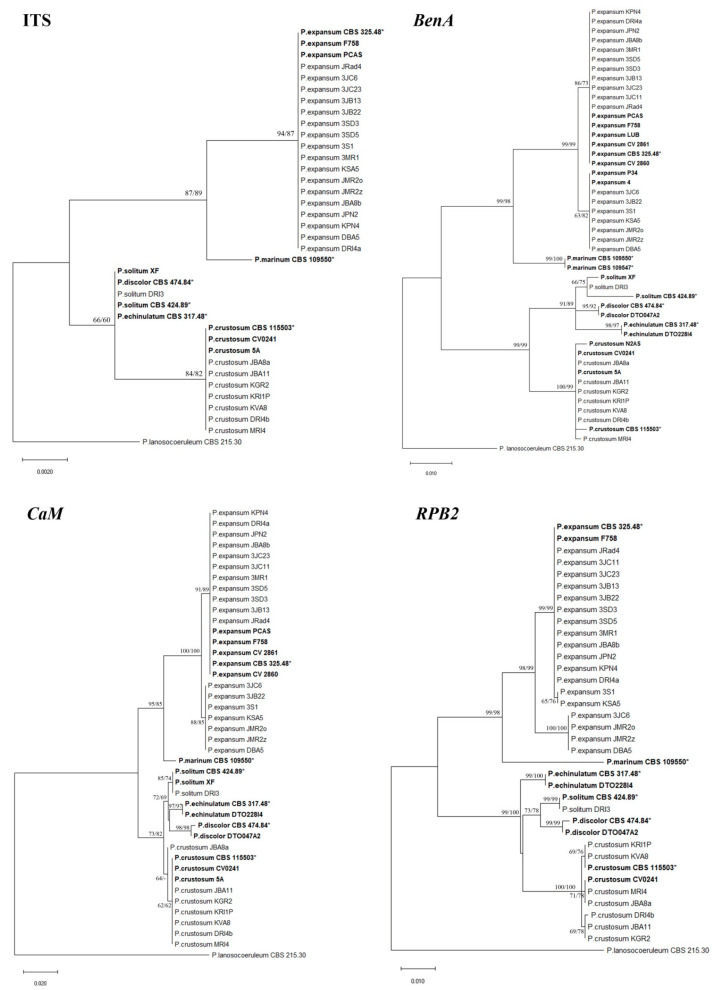
Phylogenetic relationships amongst *Penicillium expansum*, *P. crustosum*, and *P. solitum* based on ITS, *BenA*, *CaM*, and *RPB2*. Bootstrap values larger than 60 of the Maximum Likelihood (ML) and Maximum Parsimony (MP) analysis are shown above or below the branches. The tree is rooted with *Penicillium lanosocoeruleum* as an outgroup. Numbers on the branches present bootstrap values obtained for 1000 replicates. Bolded isolates are reference isolates, and bolded isolates with * are ex-type isolates.

**Figure 7:**
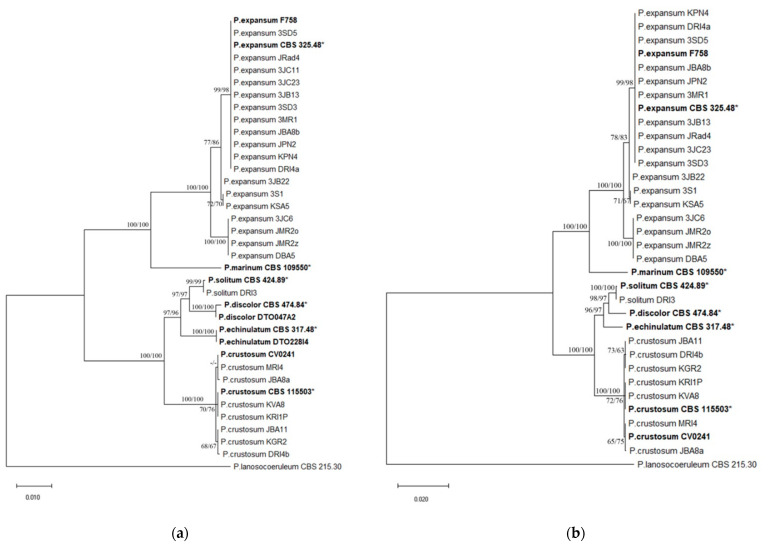
Phylogenetic relationships amongst *Penicillium expansum*, *P. crustosum*, and *P. solitum* based on three (*BenA, CaM*, and *RPB2*) (**a**) and four loci (ITS, *BenA, CaM*, and *RPB2*) (**b**). Bootstrap values larger than 60 of the Maximum Likelihood (ML) and Maximum Parsimony (MP) analysis are shown above or below the branches. The tree is rooted with *Penicillium lanosocoeruleum* as an outgroup. Numbers on the branches present bootstrap values obtained for 1000 replicates. Bolded isolates are reference isolates, and bolded isolates with * are ex-type isolates.

The ITS, *BenA*, *CaM*, and *RPB2* multiple sequence alignments contained 561, 448, 521, and 971 nucleotides, of which 9, 40, 53, and 90, were parsimony informative, respectively. Different nucleotide substitution models were used for individual ML analyses: Tamura’s 3-parameter model (T92) for ITS, Kimura’s 2-parameter model (K2) for *BenA* and *CaM*, and Kimura’s 2-parameter model with a discrete gamma distribution (K2 + G) for RPB2. MP analyses of ITS, *BenA*, *CaM*, and *RPB2* resulted each in 10 equally most parsimonious trees. ML and MP analyses, based on the same single locus, produced trees with identical topologies. Comparing the trees inferred from different loci, the most congruent were those based on *BenA* and *CaM*. The combined dataset of the concatenated three single locus alignments (*BenA*, *CaM*, and *RPB2*) contained 1928 characters, of which 176 were parsimony informative, and the combined dataset of the concatenated four single locus alignments contained 2498 characters, of which 169 were parsimony informative. Multilocus phylogenetic trees constructed by ML method, using K2 + G, had the same topology as MP trees, and were consistent with topology of *RPB2* tree. MP analysis resulted in 10 equally most parsimonious trees. Selected phylogenetic trees are presented in Figure 6 and Figure 7.

Each phylogenetic analysis conducted in this study clearly separated *P. expansum* and *P. crustosum.* No difference within species was observed in phylogenetic analysis based on the ITS region. *P. expansum* isolates were (sub) divided into two well-supported clades based on *BenA* and *CaM* phylogeny, corresponding to two groups separated by macromorphology. *RPB2* and multilocus phylogeny confirmed the separation of *P. expansum* isolates into two groups with the exception of three isolates that were relocated from one group to another (Table 5).

*P. crustosum* isolates resided in one clade based on ITS, *BenA* and *CaM* phylogeny. *RPB2* and multilocus phylogeny revealed three groups within *P. crustosum* clade, all of which received bootstrap support above 60% in ML analyses based on both datasets. *P. solitum* isolate was grouped with reference isolates of *P. solitum* into one clade based on each phylogenetic analysis conducted in this study, except the one based on ITS which could not separate *P. solitum* from some similar *Penicillium* species. *P. solitum* clade received the highest bootstrap support (100%) in multilocus phylogeny, followed by *RPB2* phylogeny (99%). *RPB2* has shown to be the most variable region in all obtained *Penicillium* spp.

### 3.8. Virulence Phenotypes in Apple Fruit

**Figure 8:**
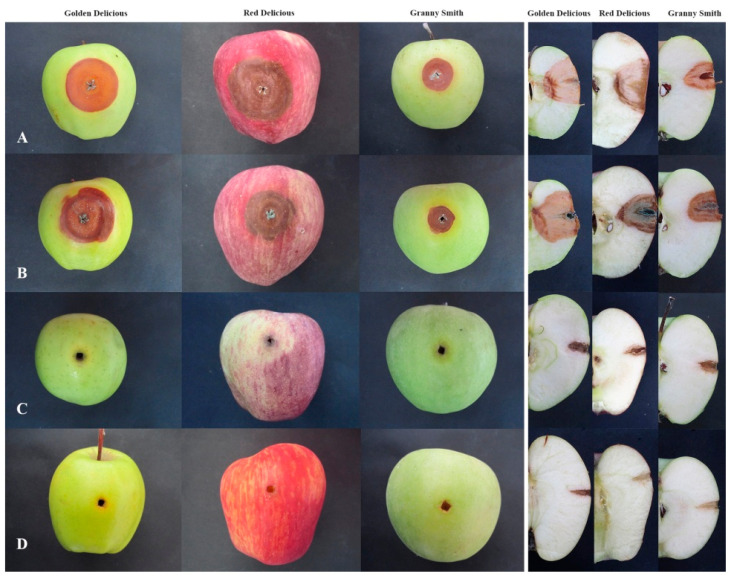
Blue mold decay caused by *Penicillium expansum* (**A**), *P. crustosum* (**B**), and *P. solitum* (**C**) on different apple fruit cvs. 9 dpi at 24 ± 2 °C and control (**D**).

Representative *P. expansum* and *P. crustosum* isolates caused typical blue mold symptoms in all apple cultivars evaluated. Lesions were soft, watery, light to medium brown, with or without concentric zones, with smooth or irregular margins, and with blue green conidial tufts present mainly around the inoculation cite. In some cases (i.e., *P. crustosum* isolates JBA8a, MRI4) on apple fruit ‘Granny Smith’ a yellow ring was observed around decayed area. On cross sections of inoculated apple fruit, differences between the decay caused by *P. expansum* and *P. crustosum*, were observed. *P. crustosum* isolates caused slightly darker color of the decayed tissue in all cultivars and sporulated both around the wound an in the apple flesh of ‘Red Delicious’. *P. expansum* isolates formed lightly coloured decayed areas and scarcely sporulated only around the inoculation cite, not in the apple flesh. In apple fruit inoculated with *P. solitum*, tissue darkening was observed only around inoculation cite and a yellow ring was present (Figure 8).

**Figure 9:**
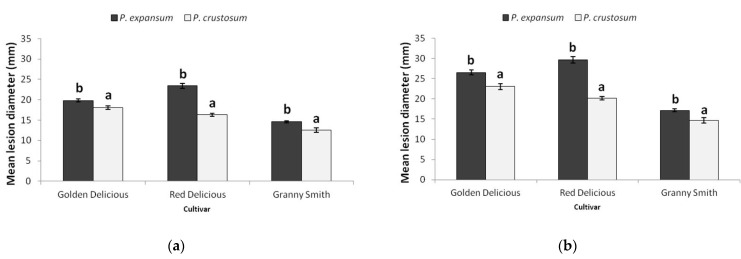
*Penicillium expansum* and *P. crustosum* mean lesion diameter (mm ± SE) on inoculated apple fruit ‘Golden Delicious’, ‘Red Delicious’ and ‘Granny Smith’ at 7 dpi (**a**) and 9 dpi (**b**). Numbers with different letters represent significant difference in lesion diameter amongst species according to Tukey HSD test (*p* < 0.01).

Based on lesion size, *P. expansum* was significantly more virulent than *P. crustosum* on all three apple cultivars (‘Golden Delicious’, ‘Red Delicious’, and ‘Granny Smith’), 7 dpi (*p* = 0.003, *p* < 0.0001, *p* = 0.001) (Figure 9a) and 9 dpi (*p* < 0.0001, *p* = 0.001, *p* = 0.002) (Figure 9b). Nevertheless, virulence of each species depended on the apple cultivar. *P. expansum* was the most virulent on ‘Red Delicious’, and produced larger lesions than on ‘Golden Delicious’ (*p* < 0.0001). *P. crustosum* was equally virulent on ‘Golden Delicious’ and ‘Red Delicious’ (7 dpi, *p* = 0.015). Both species were the least virulent on ‘Granny Smith’. The largest difference in virulence between *P. expansum* and *P. crustosum* was observed on ‘Red Delicious’.

Within species, variability in virulence was observed amongst *P. expansum* isolates on ‘Red Delicious’ (7 dpi *p* < 0.0001), and amongst *P. crustosum* isolates on ‘Granny Smith’ (7 dpi, *p* < 0.0001). Cluster analyses based on lesion size, separated three groups within *P. crustosum* isolates the same way that was determined for colony growth (shown in Figure 5), and it corresponds with three sub clusters obtained via phylogenetic analysis (*RPB2* and multilocus). No influence of isolate origin on virulence was observed (statistical data not shown). Two-way ANOVA revealed that both apple cultivar (η^2^ = 0.419 for 7 dpi and η^2^ = 0.475 for 9 dpi) and species (η^2^ = 0.237 for 7 dpi, η^2^ = 0.252 for 9 dpi) had a large influence on lesion size. Combined influence of both factors: species and apple cultivar on lesion size was moderate (η^2^ = 0.123 for 7 dpi and η^2^ = 0.110 for 9 dpi), suggesting that the combination of the most susceptible cultivar and the most virulent pathogen caused rapid disease progression (‘Red Delicious’ + *P. expansum*), while disease progressed more slowly in case of less susceptible cultivar and less aggressive pathogen (‘Granny Smith’ + *P. crustosum*).

### 3.9. Principle Component Analysis

**Figure 10:**
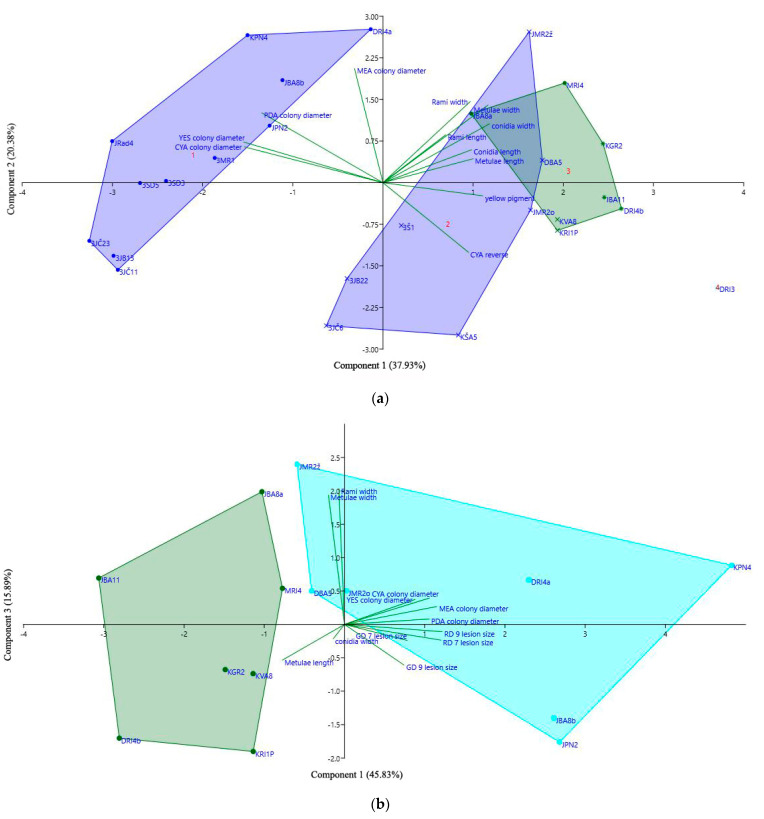
Projection of *Penicillium expansum* and *P. crustosum* isolates based on: (**a**) first and second principal component, according to principal component analysis (PCA) of isolate characteristics (micro, macromorphology, and colony growth). *P. expansum* isolate groups are purple, while *P. crustosum* isolate group is green (DRI3 is *P. solitum* isolate); (**b**) first and third principal component, according to principal component analysis (PCA) of isolate characteristics (micro, macromorphology, colony growth, and virulence). *P. expansum* isolate group is turquoise, while *P. crustosum* isolate group is green.

PCA analysis separated all 26 tested isolates based on morphological data (micro, macro and growth rate) in three groups (Figure 10a). The total variability of 58.31% was explained by first and second component. Principal component 1 (PC1), accounts for 37.93% of the variance, was mostly positively associated with conidia and metulae width and negatively associated with colony diameter on PDA, CYA and YES. It clearly separates faster growing isolates (left) from those with smaller colonies and with larger conidia or conidiophore parts (right). PC2, which accounts for 20.38% of the variance, is strongly positively associated with colony diameter on MEA and PDA, and metulae width, while is negatively associated with coloration on CYA reverse (red and pink coloration has smaller value than yellow or cream coloration). It separates faster growing isolates firstly on MEA, then on PDA with wider metulae (up) from isolates that have cream or yellow coloration on CYA reverse (down). It also shows the similarity of the second *P. expansum* group to *P. crustosum*. Separation of *P. expansum* isolates into two groups based on morphology was in accordance with phylogenetic separation based on single *BenA* and *CaM* loci (Table 5).

PCA analysis grouped 14 representative isolates based on macro- and micromorphology and virulence on ‘Red Delicious’ and ‘Golden Delicious’ at 7 and 9 dpi. The total variability of 61.72% was explained by first and third component. PC1 accounts for 45.83% of the observed variance, and was mostly associated with colony diameter on all tested media, and lesion size on ‘Red Delicious’ at 7 dpi and 9 dpi. It separates more virulent isolates with larger colony growth, from less virulent isolates, which formed smaller colonies. Thus, the majority of *P. expansum* isolates were distinct from *P. crustosum* isolates, and were grouped on the right side of the graph, except from two isolates DBA5 and JMR2z. PC3, which accounts for 15.89% of the variance, is strongly and positively associated with metulae and rami width, and separates isolates with wider metulae and rami (up) from isolates that formed narrower metulae and rami (down) (Figure 10b).

## 4. Discussion

Data regarding the incidence of blue mold decay, diversity of pome fruit hosts, variation in fungal virulence, and distribution of *Penicillium* spp. causing postharvest rot resulted in confirmatory and novel findings. *Penicillium expansum* and *P. crustosum* caused blue mold on stored apple and pears which is congruent with previous studies [20,21,22,23,24,55], while blue mold on quince caused by *P. expansum*, *P. crustosum* and *P. solitum* is a novel find in Serbia. Additionally, *P. crustosum* has not been identified on medlar nor quince, indicating two new hosts for this blue mold causing species. Occurrence of *P. solitum* on quince has not been previously reported in the literature, and multiple gaps regarding various pome fruit hosts and causative *Penicillium* spp. were unknown. Hence, novel discoveries resulted from the comprehensive survey, virulence assessment and accompanying morpho-genetic characterization of *Penicillium* spp.

Incidence regarding the most common causal agent of blue mold on pomaceus fruit in Serbia showed that *P. expansum* was most prevalent (91.64%), followed by *P. crustosum* (7.29%) which were detected on all pome fruit examined, with *P. solitum* having the lowest occurrence. A survey of apples and pears with blue mold symptoms from Oregon and Washington State showed that several *Penicillium* spp. (*P. auarantiogriseum*, *P. commune*, *P. solitum*, *P. verrucosum*, and *P. expansum*) caused blue mold [14]. Another study conducted in British Columbia showed that *P. brevicompactum*, *P. crustosum*, and *P. expansum* were isolated from apple fruit with blue mold symptoms [15]. In Uruguay, it was found that *P. expansum* and *P. solitum* were the main causal agents of apple and pear decay in storage [13]. Our findings mirror what has been shown over different parts of the world regarding *Penicillium* spp. diversity, and it appears that regardless of geographical location, *P. expansum* is most prevalent and aggressive of all blue mold fungi obtained from stored pomes. Many factors may underpin *P. expansum’s* cosmopolitan status including but not limited to: e.g., the pathogens genomic plasticity, its ability to supress, inactivate and overcome preformed and induced host plant defences, deployment of an arsenal of virulence factors, and the biosynthesis of small molecules that aid in decay [56]. Future comparative multiomics investigations with the isolates from this study will be used to ascertain the underlying mechanism(s) that facilitate *P. expansum’s* broad ranging success as a necrotrophic fungal pathogen.

*Penicillium expansum* is the most virulent *Penicillium* species and is well adapted to infect, colonize and decay apple fruit [9]. Our findings agree with Morales et al. [57] that there is low variability in *P. expansum’s* ability to colonize apple flesh, however variation in some features (e.g., macromorphology and phylogeny) was observed. Also, we support the suggestion of Morales et al. [57] that factors involved in fruit colonization seem to be constant in each *P. expansum* population. In this study *P. expansum* was found to be more virulent than *P. crustosum* on all three apple fruit cultivars, which was also observed in other studies on stored apple and pear fruits [6,14,58]. Differences in *P. expansum* and *P. crustosum* virulence in apple could be explained via metabolic flux in the host during the infection process. *P. expansum* causes more intense and dynamic metabolic changes which helps the pathogen overcome host defenses and results in decreased pools of phenolics and glutathione compared to *P. crustosum*-mediated decay [59,60]. It is well known that high phenolic content is important for apple fruit defence against blue mold decay [61,62,63]. This difference is also reflected in the array of observed virulence phenotypes in apple fruit conducted during this study. Even though *P. crustosum* is a less well adapted pathogen compared to *P. expansum*, it caused decay in apple fruit, and is supported by its ability to abundantly sporulate in the wound. Together, these factors allow *P. crustosum* to be classified as the second most important *Penicillium* species found to cause apple fruit decay. However, both *P. expansum* and *P. crustosum* were least virulent on ’Granny Smith’ and its hypothesized that this could be due to differences in flavanol and glutathione content, which is higher in this cultivar than others [64,65]. Additionally, a yellow ring surrounding the infected area was primarily observed in ’Granny Smith’ infected with *P. crustosum*. Interestingly, a similar reaction was observed on immature and commercially mature ‘Golden Delicious’ fruit infected with an incompatible apple fruit pathogen *P. digitatum* [66]. Both infection and wounding activate H_2_O_2_ accumulation to generate high ROS levels which can damage chloroplasts, resulting in the yellow-colored ring, reminiscent of the same phenomenon we observed during our study [67,68].

Since *P. expansum* and *P. crustosum* were found to be the most dominant causal agents of blue mold in Serbia, the isolates from different origins were subjected to detailed morphological characterization. We observed that YES was the most favourable media for the growth of both species, which agreed with descriptions for *P. expansum* and *P. crustosum* by Pitt and Hocking [69] and Frisvad and Samson [18]. Interestingly, two distinct groups of *P. expansum* were observed based on colony growth, presence/absence of yellow colony reverse on PDA and MEA, and CYA reverse. The first group had: faster growing colonies, cream reverse on PDA and MEA, and salmon pink to red CYA reverse while the second group had: slower colony growth, yellow reverse on PDA and MEA, and variable colour of CYA colony reverse (yellow, cream or red). Variability and correlation of morphological and genetic characteristics was observed for some other *Penicillium* species such as *P. glabrum*. Barreto et al. [70] noted high intraspecific variation of *P. glabrum* from cork in micro-and macromorphology and extrolite profiles, which was supported by partial β-tubulin and calmodulin sequence analyses. Similarly, variability of *P. glabrum* isolates from onion bulbs was observed via molecular (*BenA*), macromorphological and pathogenic characters and these features were correlated [49].

Examination of the *Penicillium* spp. isolates using multiple loci (ITS, *BenA, CaM*, *RPB2*) largely agreed with the morphological findings to reveal three main species of blue mold fungi from four different pome fruit hosts. While several distinct *Penicillium* spp. were isolated and characterized, we observed the presence of multiple, well supported subgroups for *P. expansum* based on individual and concatenated loci. Our data show that two well supported subclades, congruent with morphological data, were present for *P. expansum* via *BenA*, *CaM*, and concatenated loci. While the consequence for this observation is unclear, it is possible that *P. expansum* is undergoing genetic change(s) that accompany the observed morphological differences that function in ecological, biological and or niche specialization. Despite the biological consequence, our findings are congruent with the literature that the *BenA* is amongst the most stable and of utmost phylogenetic value for *Penicillium* speciation [50]. Houbraken et al. [42] indicate that subspecific levels such as subspecies, varieties, *forma specialis* etc. should not be used in a formal taxonomic manner. While their taxonomic value remains debatable, a deeper look into the various subspecies via comparative approaches (e.g., metabolite profiles, genomic organization, and secondary metabolic gene cluster arrangement) will likely yield an abundance of fundamental information to ascertain the biological basis for these observations.

While *P. expansum* and *P. crustosum* were the most prevalent blue mold fungi obtained from pome fruits, isolate origin did not impact the observed morphological, genetic and pathogenic features evaluated in the current study. This is in contrast with what Sanzani et al. [71] who showed genetic and morphological variation amongst *P. expansum* and *P. crustosum* isolates, based on a single locus *BenA*, which grouped the isolates based on the origin of the host (confirmed by the High-Resolution Melting). Thus, they proposed that host specialization had occurred amongst the isolates of the same species. However, we didn’t observe this pattern. It is possible that our isolates are not as evolved and or that the isolates from Italian collection possess more genetic variation at loci that mediate outcrossing and or recombination. A detailed genome-wide study conducted by Julca et al. [72] supports our finding in that they showed a relatively high sequence divergence of *P. expansum*, which did not correlate with the geographical distance between isolates. This indicates that isolates may have diverged a long time ago but that the geographical structure of the populations may have been influenced by migration. *P. expansum* has the capacity to undergo both meiotic and mitotic recombination and exhibits large genetic diversity, despite its primary asexual mode of reproduction. This is further supported by genome mining studies that show *P. expansum* is heterothallic. Hence, there is potential that cryptic mating amongst divergent strains may have contributed to the observed patterns of genomic variation [72].

Our two-year survey highlights new morphological, and phylogenetic aspects of *Penicillium* spp. from stored pome fruits in Serbia, has filled several knowledge gaps, brought forth new findings concerning fungal virulence, and uncovered interesting observations to further explore. Our study showed for the first time that *P. crustosum* and *P. solitum* infect quince, and that medlar is a new host of *P. crustosum*. Additionally, *P. expansum* is a new pathogen of quince in Serbia. Rigorous phylogenetic and morphological investigations show that there are three species that predominate pome fruit storage and that *P. expansum* has two distinct phylogenetic subgroups and *P. crustosum* has three according to various loci. Consistent with previous findings we have shown that in general, *P. expansum* is the most commonly isolated and aggressive blue mold species followed by *P. crustosum* and the least prevalent and weakest is *P. solitum.* Our morphological and phylogenetic findings suggest that *P. expansum* is changing at multiple levels and we propose future omics-based and functional genetic studies to understand the basis for the observed differences within *P. expansum* sub-clades.

## Figures and Tables

**Table 2:** Haplotype analysis of *Penicillium expansum* and *P. crustosum* isolates.

Sequence	Position														
***Penicillium expansum***															
***BenA***	232	260													
I variant	T	C													
II variant	G	T													
***CaM***	79	154	356												
I variant	T	C	A												
II variant	C	T	G												
***RPB2***	139	151	325	340	373	454	520	760	841	854	889	943	958	979	1036
I variant	C	A	G	T	G	C	T	C	T	C	T	G	A	C	C
II variant	T	G	A	C	A	T	C	T	C	C	C	A	C	T	T
III variant	C	A	G	T	G	C	T	C	T	T	T	G	A	C	C
***Penicillium crustosum***															
***CaM***	303	337	414												
I variant	C	A	T												
II variant	-	G	-												
***RPB2***	175	724	940	979											
I variant	T	C	A	C											
II variant	T	C	C	T											
III variant	T	T	C	C											
IV variant	C	T	C	C											

Gray background represents differences amongst variants.

**Table 3:** Colony diameter of *Penicillium, expansum*, *P. crustosum* and *P. solitum* isolates on different media 7 dpi, in the dark at 24 ± 2 °C.

Isolate	Colony Diameter on Different Culture Media (mm ± Standard Deviation)			
	PDA	MEA	CYA	YES
***Penicillium expansum***				
JRad4	47.00 ± 0.97f *	37.22 ± 0.73hi	48.72 ± 1.25h	51.31 ± 1.21d–f
3JC11	42.69 ± 0.86d–f	30.67 ± 1.08b–d	44.18 ± 1.59d–g	49.71 ± 1.78c–f
3JC23	45.81 ± 1.02ef	31.58 ± 0.69c–e	43.09 ± 1.10c–f	52.44 ± 0.63ef
3JB13	40.58 ± 0.81b–e	29.42 ± 1.48a–c	46.13 ± 1.09e–h	50.53 ± 0.85d–f
3SD3	43.85 ± 1.26d–f	31.06 ± 0.97c–e	46.94 ± 1.57f–h	51.69 ± 1.14d–f
3SD5	43.92 ± 0.97d–f	30.72 ± 0.89b–d	46.34 ± 1.66e–h	53.28 ± 1.17f
3MR1	44.83 ± 1.20d–f	32.08 ± 1.03de	47.73 ± 1.02gh	49.10 ± 1.54b–f
JBA8b	43.47 ± 7.05d–f	36.69 ± 2.12gh	44.39 ± 6.26e–h	49.64 ± 9.88c–f
JPN2	43.92 ± 7.85d–f	37.89 ± 1.92hi	42.42 ± 5.53b–e	48.39 ± 8.64b–f
KPN4	44.56 ± 8.03d–f	39.03 ± 3.58i	44.50 ± 5.96e–h	48.89 ± 8.77b–f
DRI4a	44.91 ± 7.60d–f	37.50 ± 2.81–i	45.14 ± 6.31e–h	50.21 ± 8.46d–f
3JC6	36.77 ± 0.87a–c	27.33 ± 0.49a	46.22 ± 0.86e–h	49.31 ± 1.01b–f
3JB22	39.33 ± 1.42b–d	28.64 ± 1.41ab	38.69 ± 0.67a–c	45.00 ± 2.61b–d
3S1	32.92 ± 1.62a	36.38 ± 1.50gh	42.47 ± 0.63b–e	48.03 ± 1.47b–f
KSA5	36.64 ± 0.70ab	27.25 ± 0.46a	38.36 ± 1.12ab	42.50 ± 1.72ab
JMR2o	35.03 ± 4.24ab	32.06 ± 1.03de	38.39 ± 3.21ab	37.53 ± 2.17a
JMR2z	42.41 ± 6.47c–f	34.75 ± 2.06fg	39.94 ± 2.43a–d	45.72 ± 6.56b–e
DBA5	36.78 ± 4.56a–c	33.17 ± 1.20ef	36.28 ± 2.15a	43.00 ± 5.54a–c
**Avg.**	41.45 ± 5.76b **	**32.94 ± 4.00b**	**43.17 ± 4.75c**	**48.03 ± 6.25c**
***Penicillium crustosum***				
JBA8a	39.58 ± 5.18a	35.03 ± 2.27c	43.81 ± 5.99b	47.19 ± 8.38ab
JBA11	36.38 ± 3.09a	30.39 ± 1.12a	36.00 ± 2.54a	41.61 ± 5.13ab
KGR2	38.36 ± 2.84a	32.39 ± 1.36ab	39.13 ± 6.09ab	44.09 ± 6.19ab
KRI1P	38.97 ± 2.61a	31.34 ± 1.57ab	38.69 ± 3.28ab	40.92 ± 4.72a
KVA8	37.91 ± 3.37a	31.64 ± 1.60ab	38.92 ± 3.79ab	41.58 ± 5.12ab
DRI4b	37.71 ± 3.07a	33.25 ± 1.40bc	35.53 ± 1.69a	41.44 ± 4.35ab
MRI4	39.71 ± 4.10a	35.26 ± 2.48c	40.53 ± 5.64ab	48.47 ± 8.46b
**Avg.**	**38.39 ± 3.64b**	**32.77 ± 2.45b**	**38.90 ± 5.05b**	**43.51 ± 6.67b**
***Penicillium solitum***				
DRI3	28.02 ± 0.50a	24.31 ± 0.68a	29.75 ± 0.69a	34.53 ± 1.22a

* Numbers with different letters represent a statistically significant difference in colony diameter within isolates of the same species per media according to Tukey HSD test (*p* < 0.01). ** Bolded numbers with different letters represent significant difference in colony diameter among species per media according to Tukey HSD test (*p* < 0.01).

**Table 4:** Micromorphology of *Penicillium expansum*, *P. crustosum* and *P. solitum* isolates.

Isolate	Average Size (µm ± Standard Deviation)							
	Conidia		Phialides		Metulae		Rami	
	Length	Width	Length	Width	Length	Width	Length	Width
***Penicillium expansum***								
JRad4	3.25 ± 0.25c–e *	2.72 ± 0.19bc	9.74 ± 1.38ab	2.84 ± 0.55a–d	12.72 ± 2.41a–e	3.35 ± 0.59a–e	19.64 ± 4.20a–c	3.67 ± 0.63ab
3JC11	3.13 ± 0.30a–d	2.56 ± 0.26ab	10.06 ± 0.91a–d	2.85 ± 0.34a–d	11.63 ± 1.46a	3.19 ± 0.38ab	18.61 ± 2.10a–c	3.58 ± 0.43a
3JC23	3.13 ± 0.24a–d	2.52 ± 0.18a	9.09 ± 1.35a	2.89 ± 0.53a–e	11.99 ± 2.65a–c	3.23 ± 0.68a–c	16.67 ± 3.51a	3.78 ± 0.77ab
3JB13	3.00 ± 0.22ab	2.52 ± 0.19a	9.82 ± 0.77ab	2.69 ± 0.28a–c	12.94 ± 1.83a–e	3.27 ± 0.36a–d	19.58 ± 2.77a–c	3.56 ± 0.39a
3SD3	3.05 ± 0.22a–c	2.54 ± 0.21ab	9.65 ± 1.05ab	2.74 ± 0.39a–d	12.77 ± 3.19a–e	3.56 ± 0.72a-g	18.55 ± 4.15a–c	4.15 ± 0.68a–c
3SD5	2.96 ± 0.23a	2.56 ± 0.20ab	10.13 ± 1.21b–d	2.97 ± 0.40b-e	11.81 ± 2.09ab	3.78 ± 0.76e-h	17.83 ± 3.16a–c	4.10 ± 0.64a–c
3MR1	3.16 ± 0.29a-d	2.56 ± 0.21ab	10.48 ± 1.11b–d	2.92 ± 0.37b-e	13.51 ± 1.76c–e	3.51 ± 0.37a-f	24.65 ± 5.84d	3.74 ± 0.39ab
JBA8b	3.22 ± 0.24b-e	2.98 ± 0.24de	9.86 ± 1.11a–c	3.03 ± 0.39d–f	14.30 ± 2.26e	3.64 ± 0.54b-g	20.45 ± 4.33a-d	4.01 ± 0.64a–c
JPN2	3.25 ± 0.31c–e	2.80 ± 0.22cd	10.36 ± 1.35b–d	2.98 ± 0.36c–e	13.50 ± 1.96b-e	3.67 ± 0.43c–h	19.39 ± 4.75a–c	3.83 ± 0.63ab
KPN4	3.27 ± 0.37c–e	2.98 ± 0.30de	10.91 ± 1.46d	3.20 ± 0.35ef	14.01 ± 2.51e	3.96 ± 0.53fgh	22.02 ± 4.64cd	4.17 ± 0.56a–c
DRI4a	3.61 ± 0.39f	3.22 ± 0.30f	9.75 ± 1.38ab	3.03 ± 0.44d–f	13.62 ± 2.06c–e	3.91 ± 0.65fgh	18.91 ± 3.56a–c	4.23 ± 0.45bc
3JC6	3.56 ± 0.27f	2.99 ± 0.29e	10.27 ± 1.05b–d	2.57 ± 0.27a	11.29 ± 1.18a	3.13 ± 0.47a	17.89 ± 2.26a–c	3.58 ± 0.54a
3JB22	3.10 ± 0.24a–d	2.47 ± 0.17a	10.33 ± 1.02b–d	2.64 ± 0.38ab	13.84 ± 2.38de	3.58 ± 0.77a–g	19.91 ± 4.39a–c	3.83 ± 0.81ab
3S1	2.93 ± 0.29a	2.48 ± 0.23a	10.39 ± 1.79b–d	2.97 ± 0.44b–e	12.79 ± 2.09a–e	3.69 ± 0.77d–h	20.92 ± 3.60b–d	4.15 ± 0.94a–c
KSA5	3.32 ± 0.29de	2.63 ± 0.19ab	10.24 ± 1.32b–d	2.79 ± 0.32a–d	12.63 ± 1.76a–e	3.36 ± 0.44a–e	19.75 ± 4.11a–c	3.83 ± 0.48ab
JMR2o	3.46 ± 0.39ef	2.82 ± 0.31c–e	9.78 ± 1.10ab	3.32 ± 0.55f	12.15 ± 1.45a–d	4.10 ± 0.47h	16.79 ± 3.22ab	4.25 ± 0.65bc
JMR2z	3.42 ± 0.36ef	2.99 ± 0.25e	10.34 ± 1.11b–d	3.20 ± 0.53ef	13.90 ± 2.45e	4.11 ± 0.60h	21.75 ± 5.48cd	4.58 ± 0.53c
DBA5	3.45 ± 0.47ef	2.81 ± 0.31c–e	10.83 ± 1.47cd	3.20 ± 0.52ef	13.46 ± 2.28b–e	4.01 ± 0.53gh	20.61 ± 5.60a–d	4.26 ± 0.53bc
**Avg.**	3.24 ± 0.30a **	**2.73 ± 0.24a**	**10.14 ± 1.32a**	**2.94 ± 0.47a**	**12.94 ± 2.30a**	**3.61 ± 0.65a**	**19.66 ± 4.47ab**	**3.96 ± 0.97a**
***Penicillium crustosum***								
JBA8a	3.10 ± 0.24a	2.76 ± 0.23a	10.67 ± 1.56d	3.08 ± 0.49a	14.37 ± 2.30ab	4.10 ± 0.53b	21.52 ± 4.38ab	4.31 ± 0.69b
JBA11	3.27 ± 0.38a–c	3.00 ± 0.27b	10.59 ± 1.52cd	3.23 ± 0.51a	13.30 ± 11.86a	4.01 ± 0.56ab	20.37 ± 3.65ab	4.22 ± 0.57ab
KGR2	3.61 ± 0.27d	3.34 ± 0.22c	9.67 ± 1.05a–c	3.03 ± 0.32a	13.71 ± 1.88ab	3.71 ± 0.46a	21.18 ± 3.87ab	4.16 ± 0.44ab
KRI1P	3.28 ± 0.23a–c	3.06 ± 0.18b	10.36 ± 1.57b–d	3.09 ± 0.34a	13.97 ± 2.79ab	3.77 ± 0.53a	20.01 ± 4.73a	3.88 ± 0.48ab
KVA8	3.23 ± 0.21ab	2.97 ± 0.20b	9.63 ± 1.10ab	3.06 ± 0.38a	13.62 ± 3.05a	3.83 ± 0.48ab	19.87 ± 4.55a	4.10 ± 0.66ab
DRI4b	3.36 ± 0.33bc	2.98 ± 0.27b	9.36 ± 0.96a	2.99 ± 0.39a	15.04 ± 2.24b	3.75 ± 0.54a	23.37 ± 4.33b	3.78 ± 0.59a
MRI4	3.45 ± 0.23c	3.28 ± 0.23c	10.07 ± 1.41a–d	3.21 ± 0.45a	14.34 ± 1.79ab	3.86 ± 0.58ab	22.26 ± 4.82ab	4.28 ± 0.55b
**Avg.**	**3.33 ± 0.27a**	**3.06 ± 0.23b**	**10.05 ± 1.35a**	**3.10 ± 0.41a**	**14.02 ± 2.43b**	**3.86 ± 0.54b**	**21.23 ± 4.45b**	**4.11 ± 0.60ab**
***Penicillium solitum***								
DRI3	3.67 ± 0.17b	3.42 ± 0.18c	10.13 ± 1.92a	3.11 ± 0.41a	12.20 ± 2.03a	4.21 ± 0.56c	18.17 ± 4.09a	4.41 ± 0.69b

* Numbers with different letters represent significant difference within isolates of the same species according to Tukey HSD test (*p* < 0.01); ** Bolded numbers with different letters represent significant difference in micromorphology among species according to Tukey HSD test (*p* < 0.01).

**Table 5:** Placement of *Penicillium expansum* isolates based on different characteristics.

Macromorphology (Cluster Analysis: Colony Diameter, PDA, MEA, CYA Reverse)		Phylogeny	
		*BenA* *CaM*	*RPB2* Multilocus
I group	JRad4 *	**JRad4**	**JRad4**
	**3JC11**	**3JC11**	**3JC11**
	**3JC23**	**3JC23**	**3JC23**
	**3JB13**	**3JB13**	**3JB13**
	**3SD3**	**3SD3**	**3SD3**
	**3SD5**	**3SD5**	**3SD5**
	**3MR1**	**3MR1**	**3MR1**
	**JBA8b**	**JBA8b**	**JBA8b**
	**JPN2**	**JPN2**	**JPN2**
	**KPN4**	**KPN4**	**KPN4**
	**DRI4a**	**DRI4a**	**DRI4a**
			3JB22
			3S1
			KSA5
II group	3JB22	3JB22	
	3S1	3S1	
	KSA5	KSA5	
	**3JC6**	**3JC6**	**3JC6**
	**JMR2o**	**JMR2o**	**JMR2o**
	**JMR2z**	**JMR2z**	**JMR2z**
	**DBA5**	**DBA5**	**DBA5**

^*^ Bolded isolates show stable placement in groups based on all characters.

## Data Availability

All relevant data are within the manuscript. Sequence data have been uploaded on Genbank with accession numbers as indicated in Table 1.

## Supplementary Materials

The following are available online at https://www.mdpi.com/article/10.3390/jof7121019/s1, Table S1: List of *Penicillium* spp. isolates obtained in this study.
