# Supplementary material for: Incidence, Speciation, and Morpho-Genetic Diversity of Penicillium spp. Causing Blue Mold of Stored Pome Fruits in Serbia
Source: J Fungi (Basel). 2021 Nov 28;7(12):1019. doi: 10.3390/jof7121019 (PMC8709240; doi:10.3390/jof7121019)
Supplement: Supplementary file 1 [file jof-07-01019-s001.zip › jof-1470907-supplementary.pdf]

Supplementary Table S1. List of *Penicillium* spp. isolates obtained in this study

	Species	Isolate	Geographic origin	Host	Cultivar	Date
1.	***P. expansum******	**JRad4**	**Radmilovac**	**Apple fruit**	**Gloster**	**December, 2014**
2.	*P. expansum*	JRad5	Radmilovac	Apple fruit	Gloster	December, 2014
3.	*P. expansum*	JRad6	Radmilovac	Apple fruit	Gloster	December, 2014
4.	*P. expansum*	JRad7	Radmilovac	Apple fruit	Gloster	December, 2014
5.	*P. expansum*	JRad8	Radmilovac	Apple fruit	Gloster	December, 2014
6.	*P. expansum*	JRad9	Radmilovac	Apple fruit	Gloster	December, 2014
7.	*P. expansum*	JRad10	Radmilovac	Apple fruit	Gloster	December, 2014
8.	*P. expansum*	JRad11	Radmilovac	Apple fruit	Gloster	December, 2014
9.	*P. expansum*	JRad12	Radmilovac	Apple fruit	Gloster	December, 2014
10.	*P. expansum*	3JČ3/1	Čelarevo	Apple fruit	Braeburn	January, 2015
11.	*P. expansum*	3JČ3/2	Čelarevo	Apple fruit	Braeburn	January, 2015
12.	***P. expansum***	**3JČ6**	**Čelarevo**	**Apple fruit**	**Braeburn**	**January, 2015**
13.	*P. expansum*	3JB2/1	Brestovik	Apple fruit	Golden Delicious	January, 2015
14.	*P. expansum*	3JB2/2	Brestovik	Apple fruit	Golden Delicious	January, 2015
15.	*P. expansum*	3JB3	Brestovik	Apple fruit	Golden Delicious	January, 2015
16.	*P. expansum*	3JB4/1	Brestovik	Apple fruit	Golden Delicious	January, 2015
17.	*P. expansum*	3JB4/2	Brestovik	Apple fruit	Golden Delicious	January, 2015
18.	*P. expansum*	3JB5	Brestovik	Apple fruit	Jonagold	January, 2015
19.	*P. expansum*	3JB7	Brestovik	Apple fruit	Jonagold	January, 2015
20.	*P. expansum*	3JB10	Brestovik	Apple fruit	Golden Delicious	January, 2015
21.	*P. expansum*	3JB11	Brestovik	Apple fruit	Golden Delicious	January, 2015
22.	*P. expansum*	3JB12	Brestovik	Apple fruit	Golden Delicious	January, 2015
23.	***P. expansum***	**3JB13**	**Brestovik**	**Apple fruit**	**Jonagold**	**January, 2015**
24.	*P. expansum*	3JB16	Brestovik	Apple fruit	Mutsu	January, 2015
25.	*P. expansum*	3JB17	Brestovik	Apple fruit	Mutsu	January, 2015
26.	*P. expansum*	3JB18	Brestovik	Apple fruit	Mutsu	January, 2015
27.	*P. expansum*	3JB19	Brestovik	Apple fruit	Mutsu	January, 2015
28.	*P. expansum*	3JB20	Brestovik	Apple fruit	Mutsu	January, 2015
29.	***P. expansum***	**3JB22**	**Brestovik**	**Apple fruit**	**Golden Delicious**	**January, 2015**
30.	*P. expansum*	3JB23	Brestovik	Apple fruit	Golden Delicious	January, 2015
31.	*P. expansum*	3SM1	Sremska Mitrovica	Apple fruit	Jonagored	January, 2015
32.	*P. expansum*	3SD1	Smederevo	Apple fruit	Gloster	January, 2015
33.	*P. expansum*	3SD2	Smederevo	Apple fruit	Gloster	January, 2015
34.	***P. expansum***	**3SD3**	**Smederevo**	**Apple fruit**	**Red Delicious**	**January, 2015**
35.	*P. expansum*	3SD4	Smederevo	Apple fruit	Red Delicious	January, 2015
36.	***P. expansum***	**3SD5**	**Smederevo**	**Apple fruit**	**Red Delicious**	**January, 2015**
37.	*P. expansum*	3SK1	Sremski Karlovci	Apple fruit	Granny Smith	January, 2015
38.	*P. expansum*	3SK2	Sremski Karlovci	Apple fruit	Granny Smith	January, 2015
39.	***P. expansum***	**3Š1**	**Šid**	**Apple fruit**	**Red Delicious**	**January, 2015**
40.	*P. expansum*	3Š2	Šid	Apple fruit	Red Delicious	January, 2015
41.	*P. expansum*	3KAM1	Kamendol	Apple fruit	Golden Delicious	January, 2015
42.	*P. expansum*	3KAM2	Kamendol	Apple fruit	Golden Delicious	January, 2015
43.	***P. expansum***	**3MR1**	**Mala Remeta**	**Apple fruit**	**Golden Delicious**	**January, 2015**
44.	***P. expansum***	**KŠA5**	**Šabac**	**Pear fruit**	**Passe Crassane**	**March, 2015**
45.	*P. expansum*	3JČ8	Čelarevo	Apple fruit	Granny Smith	April, 2015
46.	*P. expansum*	3JČ10	Čelarevo	Apple fruit	Granny Smith	April, 2015
47.	***P. expansum***	**3JČ11**	**Čelarevo**	**Apple fruit**	**Granny Smith**	**April, 2015**
48.	***P. expansum***	**3JČ23**	**Čelarevo**	**Apple fruit**	**Modi**	**April 2015**

49.	*P. expansum*	JGR1P	Grocka	Apple fruit	Budimka	October, 2015
50.	*P. expansum*	JGR5	Grocka	Apple fruit	Idared	October, 2015
51.	***P. crustosum***	**KGR2**	**Grocka**	**Pear fruit**	**Santa Maria**	**October, 2015**
52.	*P. expansum*	DRA1P	Ralja	Quince fruit	Leskovačka	October, 2015
53.	***P. expansum***	**JMR2o**	**Mala Remeta**	**Apple fruit**	**Fuji**	**November, 2015**
54.	***P. expansum***	**JMR2ž**	**Mala Remeta**	**Apple fruit**	**Fuji**	**November, 2015**
55.	*P. expansum*	JLE1	Leskovac	Apple fruit	Idared	November, 2015
56.	*P. expansum*	JLE6	Leskovac	Apple fruit	Jonagold	November, 2015
57.	***P. expansum***	**JPN2**	**Paraćin**	**Apple fruit**	**Idared**	**December, 2015**
58.	*P. expansum*	JPN3	Paraćin	Apple fruit	Idared	December, 2015
59.	*P. expansum*	JPN5	Paraćin	Apple fruit	Golden Delicious	December, 2015
60.	*P. expansum*	JPN6	Paraćin	Apple fruit	Golden Delicious	December, 2015
61.	*P. expansum*	JPN7	Paraćin	Apple fruit	Golden Delicious	December, 2015
62.	*P. expansum*	JPN8	Paraćin	Apple fruit	Jonagold	December, 2015
63.	*P. expansum*	KPN1	Paraćin	Pear fruit	Poire de Cure	December, 2015
64.	*P. expansum*	KPN3	Paraćin	Pear fruit	Poire de Cure	December, 2015
65.	***P. expansum***	**KPN4**	**Paraćin**	**Pear fruit**	**Poire de Cure**	**December, 2015**
66.	*P. expansum*	KPN7	Paraćin	Pear fruit	Poire de Cure	December, 2015
67.	*P. expansum*	KVA1	Valjevo	Pear fruit	Poire de Cure	December, 2015
68.	*P. expansum*	KVA2	Valjevo	Pear fruit	Poire de Cure	December, 2015
69.	***P. crustosum***	**KVA8**	**Valjevo**	**Pear fruit**	**Poire de Cure**	**December, 2015**
70.	*P. expansum*	KVA9	Valjevo	Pear fruit	Poire de Cure	December, 2015
71.	*P. expansum*	KVA10P	Valjevo	Pear fruit	Poire de Cure	December, 2015
72.	*P. expansum*	KVA11	Valjevo	Pear fruit	Poire de Cure	December, 2015
73.	*P. expansum*	JBA2	Bavanište	Apple fruit	Mutsu	December, 2015
74.	*P. expansum*	JBA6	Bavanište	Apple fruit	Jonagored	December, 2015
75.	*P. expansum*	JBA7	Bavanište	Apple fruit	Jonagored	December, 2015
76.	***P. crustosum***	**JBA8a**	**Bavanište**	**Apple fruit**	**Jonagored**	**December, 2015**
77.	***P. expansum***	**JBA8b**	**Bavanište**	**Apple fruit**	**Jonagored**	**December, 2015**
78.	*P. expansum*	JBA10	Bavanište	Apple fruit	Golden Delicious	December, 2015
79.	***P. crustosum***	**JBA11**	**Bavanište**	**Apple fruit**	**Šifra**	**December, 2015**
80.	*P. expansum*	JBA13	Bavanište	Apple fruit	Idared	December, 2015
81.	*P. expansum*	JBA14	Bavanište	Apple fruit	Idared	December, 2015
82.	*P. expansum*	JBA15	Bavanište	Apple fruit	Idared	December, 2015
83.	*P. expansum*	JBA16	Bavanište	Apple fruit	Idared	December, 2015
84.	*P. expansum*	DBA3	Bavanište	Quince fruit	Leskovačka	December, 2015
85.	*P. expansum*	DBA4	Bavanište	Quince fruit	Leskovačka	December, 2015
86.	***P. expansum***	**DBA5**	**Bavanište**	**Quince fruit**	**Leskovačka**	**December, 2015**
87.	*P. expansum*	DBA7	Bavanište	Quince fruit	Leskovačka	December, 2015
88.	*P. expansum*	DBA8	Bavanište	Quince fruit	Leskovačka	December, 2015
89.	***P. crustosum***	**KRI1P**	**Ritopek**	**Pear fruit**	**Williams**	**December, 2015**
90.	***P. solitum***	**DRI3**	**Ritopek**	**Quince fruit**	**Leskovačka**	**December, 2015**
91.	***P. expansum***	**DRI4a**	**Ritopek**	**Quince fruit**	**Leskovačka**	**December, 2015**
92.	***P. crustosum***	**DRI4b**	**Ritopek**	**Quince fruit**	**Leskovačka**	**December, 2015**
93.	***P. crustosum***	**MRI4**	**Ritopek**	**Medlar fruit**	**ND**	**December, 2015**
94.	*P. expansum*	KBE1	Belosavci	Pear fruit	Poire de Cure	December, 2015
95.	*P. expansum*	JSPL2	Balta Berilovac	Apple fruit	Jonathan	January, 2016
96.	*P. expansum*	DBĆ	Bogatić	Quince fruit	Leskovačka	January, 2016

* Bolded isolates were chosen for further research
